# Impact of the new EASO obesity definition on the detection of atheromatosis in subjects with low-to-moderate cardiovascular risk

**DOI:** 10.3389/fendo.2025.1689960

**Published:** 2025-10-10

**Authors:** Josep León-Mengíbar, Marcelino Bermúdez-López, José Manuel Valdivielso, Reinald Pamplona, Gerard Torres, Dídac Mauricio, Eva Castro-Boqué, Elvira Fernández, Assumpta Caixàs, Marta Bueno, Andreea Ciudin, Marta Hernández, Rafael Simó, Cristina Hernández, Albert Lecube

**Affiliations:** ^1^ Department of Endocrinology and Nutrition, University Hospital Arnau de Vilanova de Lleida, Obesity, Diabetes and Metabolism (ODIM) Research Group, Institut de Recerca Biomèdica de Lleida (IRBLleida), University of Lleida, Lleida, Spain; ^2^ Vascular and Renal Translational Research Group, Institut de Recerca Biomèdica de Lleida (RBLleida), Renal Research Network (RICORS2040, ISCIII), Lleida, Spain; ^3^ Department of Experimental Medicine, University of Lleida, Lleida, Spain; ^4^ Department of Experimental Medicine, University of Lleida, Institut de Recerca Biomèdica de Lleida (IRBLleida), Lleida, Spain; ^5^ Translational Research in Respiratory Medicine, University Hospital Arnau de Vilanova and Santa Maria, Institut de Recerca Biomèdica de Lleida (IRBLleida), CIBER of Respiratory Diseases (CIBERES), Institute of Health Carlos III, Lleida, Spain; ^6^ Department of Endocrinology and Nutrition, Hospital de la Santa Creu i Sant Pau, CIBERDEM (Institute of Health Carlos III), Autonomous University of Barcelona, Barcelona, Spain; ^7^ Department of Endocrinology and Nutrition, Parc Taulí Hospital Universitari, Institut d’Investigació i Innovació Parc Taulí (IPT-CERCA), Medicine Department, Universitat Autònoma de Barcelona, Sabadell, Spain; ^8^ Department Endocrinology and Nutrition, University Hospital Vall d’Hebron, Diabetes and Metabolism Research Group, Institut de Recerca Vall d’Hebron (VHIR), CIBERDEM (Institute of Health Carlos III), Barcelona, Spain

**Keywords:** obesity, central adiposity, subclinical atheromatous disease, cardiovascular risk factors, waist-to-height ratio

## Abstract

**Background:**

Traditional body mass index (BMI) does not adequately reflect adipose tissue distribution and associated cardiovascular (CV) risk. To improve risk stratification, the European Association for the Study of Obesity (EASO) proposes to extend the diagnosis of obesity to include individuals with a BMI of 25–30 kg/m², a waist-to-height ratio (WtHR) ≥0.5, and any obesity-related complication.

**Objective:**

To examine whether this new definition of obesity can better identify the presence of subclinical atheromatosis disease (SAD) in terms of arterial plaque burden compared to the classical BMI-based definition.

**Methods:**

A cross-sectional including 8,330 participants from the ILERVAS project (ClinicalTrials.gov Identifier: NCT03228459), aged 45–70 years with low-to-moderate CV risk and no previous CV disease, was included. Obesity was classified using traditional (BMI ≥ 30 kg/m²) and new definitions. Atherosclerosis was assessed through Doppler ultrasound of carotid and femoral arteries. Logistic regression models adjusted for cardiovascular risk factors were used to evaluate associations between obesity definitions and SAD.

**Results:**

The new definition increased obesity prevalence from 37.2% to 71.7%. It also revealed higher detection of atheromatous plaques (72.9% vs. 68.6%, *p* < 0.001) and affected more vascular territories. Multivariable analysis showed the new definition independently predicted overall plaque presence (OR 1.54, 95% CI 1.22–1.94, *p* < 0.001) and femoral atherosclerosis (OR 1.36, 95% CI 1.10–1.68, *p* = 0.004). Similar results were obtained when only WtHR was considered, excluding obesity-related complications.

**Conclusion:**

The new obesity definition identifies more efficiently individuals at risk for atherosclerosis, especially in the femoral region, compared to the classic BMI definition. Further studies to assess the cost-effectiveness of this approach seem warranted.

## Introduction

Obesity is increasingly recognized as a complex, multifactorial, chronic, non-communicable, and relapsing disease with profound implications for both individual health and public healthcare systems worldwide (1). Despite its well-established role in the development of numerous non-communicable diseases, the clinical approach to diagnosing and managing obesity has not yet been fully aligned with the standardized processes applied to other chronic conditions (2–4). Recent evidence supports the conceptual shift from “obesity” to “obesities,” underlining that not only the amount of adipose tissue but also its distribution and functional characteristics are critical determinants in the pathophysiology of the disease and in guiding personalized therapeutic approaches (2, 4).

Body mass index (BMI) has been the cornerstone of obesity assessment in the last decades (5, 6). However, growing evidence suggests that BMI alone is insufficient to accurately reflect adipose tissue distribution or its metabolic health consequences (4, 7). While BMI-based thresholds guide diagnosis and treatment, they fail to account for individual differences in fat distribution and metabolic function (5–7). Consequently, individuals with the same BMI may exhibit markedly different metabolic profiles and health risks, with some classified as having obesity showing minimal cardiometabolic impairment, and others, despite a BMI < 30 kg/m^2^, experiencing severe obesity-related complications due to excess visceral adipose tissue (VAT). These discrepancies underscore the need to move beyond BMI-centric diagnostic models (2, 8). Accordingly, experts now advocate for the inclusion of complementary anthropometric measures, such as waist circumference, waist-to-height ratio (WtHR), and body composition metrics to improve risk stratification and clinical decision making (8, 9).

Visceral adiposity has emerged as a key contributor to cardiometabolic risk, independently of total body weight (9). VAT is metabolically active and by secreting free fatty acids and adipokines such as leptin, TNF-α, and IL-6 contributes to fostering systemic inflammation, endothelial dysfunction, and insulin resistance (10–13). These pathophysiological changes provide a mechanistic link between visceral adiposity and cardiovascular disease (CVD). In this context, atherosclerotic plaque formation represents a key clinical manifestation of VAT-driven vascular damage. Although plaque development is central to the progression of CVD, its pathogenesis is complex and appears to vary across vascular territories depending on the dominant underlying risk factors (14, 15).

In line with this updated understanding, the European Association for the Study of Obesity (EASO) has proposed a revised framework for the diagnosis, staging, and management of obesity, aligning with the current understanding of obesity as an adiposity-based chronic disease (ABCD) (4). This framework expands the diagnostic threshold beyond the traditional BMI ≥30 kg/m² to include individuals with BMI between 25 and 30 kg/m², a WtHR ≥ 0.5 and the presence of any obesity-related complication. This could allow for a more accurate identification of individuals at risk for obesity-related complications due to central adiposity accumulation (2).

In this context, our study aims to compare the predictive value of this revised definition of obesity with the traditional BMI-based criterion in identifying the presence of atheromatous plaque across different vascular territories in a large cohort of individuals at low to moderate cardiovascular (CV) risk from the province of Lleida, Spain. By integrating a more refined assessment of central adiposity, this approach may improve CV risk stratification, thus leading to more effective preventive strategies.

## Methods

### Study population, metabolic status, and patient selection

The ongoing prospective ILERVAS study (ClinicalTrials.gov Identifier: NCT03228459) investigates the progression of subclinical atherosclerotic disease (SAD) in individuals with low to moderate cardiovascular risk (16, 17). However, for the present manuscript, only baseline data were analyzed, and therefore the study design corresponds to a cross-sectional analysis. A total of 8,330 participants were initially recruited between January 2015 and December 2017 from 30 primary healthcare centers in Lleida, Spain. The inclusion criteria were as follows: participants were aged between 45 and 70 years and had no prior history of CVD, including angina, myocardial infarction, stroke, peripheral artery disease, heart failure, or any vascular surgery/procedure. Additionally, they had to present at least one cardiovascular risk factor, such as dyslipidemia, arterial hypertension, a BMI ≥ 30 kg/m², smoking, or a first-degree relative with premature CVD (myocardial infarction, stroke, or peripheral arterial disease) before the age of 55 years in men or 65 years in women. Individuals with any form of diabetes mellitus, chronic kidney disease, active neoplasia, a life expectancy of less than 18 months, or pregnancy were excluded.

Height and body weight were measured with participants wearing light clothing and without shoes. Waist circumference was measured in a standing position with a flexible tape positioned midway between the hip bone and the lower rib, and recorded to the nearest 0.1 cm. The classical definition of obesity was defined as a BMI ≥ 30 kg/m². Conversely, the new definition of obesity also included those participants with a BMI between 25 and 30 kg/m² who exhibited a WtHR ≥ 0.5 and at least one obesity-related health condition such as dyslipidemia, arterial hypertension, prediabetes, or sleep apnea (2).

Total cholesterol levels (mg/dl) were assessed in all participants using a non-fasting dried capillary blood sample (obtained via fingertip puncture) analyzed with the REFLOTRON^®^ Plus system (Roche Diagnostics, GmbH, Germany) (16, 17). A complete lipid profile, including HDL cholesterol, LDL cholesterol, and triglycerides, was measured only in participants whose total cholesterol levels ≥ 200 mg/dl after fasting for at least 6 hours, or ≥ 250 mg/dl regardless of fasting duration. Blood pressure was measured three times using an automated device [Omron M6 Comfort HEM-7221-E (Omron Healthcare, Kyoto, Japan)] after participants rested for 5 minutes. The measurements, spaced two minutes apart, were averaged from the last two readings to calculate the mean. Pulse pressure was determined as the difference between systolic and diastolic blood pressures. Prediabetes status was defined following current American Diabetes Association guidelines (18). The prevalence of dyslipidemia and hypertension were determined based on patients who were assigned a diagnostic code according to the International Classification of Diseases (ICD) Code. Smoking status was documented as non-smoker, current smoker, or former smoker, the latter defined as participants who had quit smoking for at least one year.

Prescribed treatments for hypertension and lipid management were extracted from prescription records and pharmacy invoicing data provided by the Catalan Health Service. This information is annually consolidated into the Information System for Research in Primary Care (SIDIAP) database. Antihypertensive therapies included ACE inhibitors, diuretics, angiotensin receptor blockers, beta-blockers, calcium channel blockers, and other antihypertensive agents. Lipid-lowering medications encompassed statins, fibrates, ezetimibe, and omega-3 fatty acids.

### Assessment of atheromatous plaque burden by ultrasound study

After having patients classified based on BMI or the new criteria proposed by EASO an ultrasound study was performed. The bilateral carotid arteries (common, bifurcation, internal, and external) and femoral arteries (common and superficial) were examined. Images were acquired using a Vivid-I Doppler Ultrasound system (General Electric Healthcare, Waukesha, WI, USA) equipped with a 12L-RS broadband linear probe operating at frequencies between 5 and 13 MHz. Standardized and validated protocols for image acquisition and interpretation, based on the guidelines from the International Society of Ultrasound in Medicine and Biology, were employed to minimize inter-operator variability and reduce type II errors (19). Sonographers, specifically trained in vascular ultrasound techniques with at least five years of experience in similar studies, were selected to ensure consistency in image quality. These professionals remained blinded to the participants’ clinical histories to avoid potential biases during the evaluation.

Subclinical atheromatosis was defined as the presence of any plaque in the twelve evaluated vascular regions (20). A plaque was well-defined when the focal intima-media thickness was ≥1.5 mm, projecting into the arterial lumen (21). All detected plaques were measured, and the total plaque area (cm²) was calculated using a standardized quantitative method (22).

### Statistical analysis

The normality of continuous variables was assessed using the Shapiro-Wilk test and visual inspection of quantile–quantile plots. Owing to the non-normal distribution of the data detected, quantitative dates were expressed as the medians with interquartile ranges. Categorical variables were reported as absolute frequencies. Group comparisons were conducted using Pearson’s chi-squared test for categorical variables and the Mann-Whitney U test for quantitative variables.

Univariable logistic regression was used to assess the presence of obesity in relation to SAD. Additionally, we employed a multivariable bidirectional stepwise logistic and lineal regression model to assess the presence of SAD and the number of plaques, adjusting for confounding factors such as sex, age, prediabetes status, total cholesterol, triglycerides, hypertension (yes/no), obesity (yes/no), pulse pressure, and smoking. The probability thresholds for entering and removing variables from the model were set at ≤0.05 and ≥0.10, respectively. Further, a multivariable stepwise linear regression model was applied to evaluate the total plaque area, incorporating the same set of confounders. Both logistic and linear regression models were performed separately for the classical and new definitions of obesity. Since several obesity-related complications included in the new obesity classification are actually risk factors for atherosclerosis development, a third analysis was also conducted only considering WtHR ≥ 0.5. This approach permitted us to better explore the genuine relationship between central adiposity and the presence of SAD.

The outcomes of the regression analyses were presented as odds ratios (OR) and beta coefficients with 95% confidence intervals (CIs) for logistic and linear models, respectively. Model calibration and discrimination were assessed using the Hosmer-Lemeshow goodness-of-fit test and the area under the receiver operating characteristic curve, respectively. The goodness-of-fit for the linear regression models was evaluated using adjusted *R*². All statistical analyses were conducted using STATA software version 16 and R Statistical Software version 4.1.2, with a significance threshold set at 0.05 (23).

### Ethical considerations

The ILERVAS study protocol received approval from the Ethics Committee of the University Hospital Arnau de Vilanova (initial approval: CEIC-1410, 19/12/2014). All participants provided written informed consent prior to inclusion in the study. The research was conducted in accordance with the ethical principles established in the Declaration of Helsinki and adhered to Spain’s data protection regulations.

## Results

The main clinical and metabolic characteristics of the study participants classified according to both the classical and the new definition of obesity are detailed in **Tables 1**, **2**. Based on the classical definition, 37.2% of participants were classified as having obesity. This prevalence increased significantly to 71.7% under the new definition. Individuals classified as having obesity by either definition exhibited a higher percentage of prediabetes and higher baseline blood glucose levels, presented higher rates of hypertension and pulse pressure, and were more frequently non-smokers. Notably, participants identified as having obesity under the new definition also showed a more atherogenic lipid profile and a higher prescription rate of lipid-lowering therapies compared to those not classified as obese. When participants with overweight (BMI 25–30 kg/m²) and abdominal obesity (WtHR ≥ 0.5) were included, regardless of the presence of adiposity-related comorbidities, the prevalence of obesity increased to 78.8% in our cohort, while maintaining the main clinical and metabolic characteristic described above (**Table 3**).

**Table 1:** Baseline clinical and metabolic characteristics, and atheromatous status of participants based on the classical definition of obesity (BMI ≥ 30 kg/m²).

Characteristic	No obesity group	Classical definition of obesity	*p*
*n* (%)	5.230 (62.8)	3100 (37.2)	–
Women (%)	51.1	49.9	0.292
Age (yrs)	57.5 (52.5–62.5)	58.5 (53.5–63.5)	< 0.001
BMI (kg/m²)	26.3 (24.2–28.1)	33.0 (31.3–35.8)	< 0.001
Waist to Height ratio	0.5 (0.5–0.6)	0.6 (0.6–0.7)	< 0.001
Prediabetes (%)	28.5	44.5	< 0.001
Glycaemia (mg/dl)	94.0 (88.0–101.0)	99.0 (92.0–108.0)	< 0.001
Dyslipidaemia (%)	53.9	47.4	< 0.001
Total cholesterol (mg/dl)	218.0 (193–243)	212.0 (187.0–236.0)	< 0.001
LDL-cholesterol ^a^ (mg/dl)	134.0 (113.2–156.8)	127.9 (110.4–150.0)	< 0.001
HDL-cholesterol ^a^ (mg/dl)	57.0 (49.0–67.0)	52.0 (45.0–60.0)	< 0.001
Triglycerides ^a^ (mg/dl)	108.0 (81.0–154.0)	133.0 (98.0–180.0)	< 0.001
Lipid-lowering agents (%)	17.7	18.7	0.241
Hypertension (%)	32.9	51.9	< 0.001
Systolic BP (mm Hg)	127.0 (117.0–139.0)	134.0 (124.0–145.0)	< 0.001
Diastolic BP (mm Hg)	80.0 (74.0–86.0)	84.0 (78.0–90.0)	< 0.001
Pulse Pressure (mm Hg)	47 (40.0–55.0)	49.0 (42.0–58.0)	< 0.001
Antihypertensives (%)	33.4	52.7	< 0.001
Current or former smoker (%)	47.0	34.3	< 0.001
Characteristics of atheromatous disease			
Presence of any plaque ^b^ (%)	71.8	71.4	0.710
Number of affected territories, *n*	2.0 (0.0–3.0)	2.1 (0.0–3.0)	0.660
Total plaque area (cm²)	0.5 (0.2–1.0)	0.5 (0.2–1.1)	0.744
Carotid territory affected ^b^ (%)	49.8	51.6	0.105
Number of carotid plaques, *n*	1.0 (0.0–2.0)	1.1 (0.0–2.0)	0.060
Carotid plaque area (cm²)	0.2 (0.11–0.4)	0.21 (0.11–0.42)	0.272
Femoral territory affected ^b^ (%)	55.8	54.1	0.118
Number of femoral plaques, *n*	1.0 (0.0–2.0)	1.0 (0.0–2.0)	0.305
Femoral plaque area (cm²)	0.5 (0.2–0.9)	0.5 (0.2–1.0)	0.116

Data are expressed as median (interquartile range), n, or percentage. BMI, body mass index; LDL, low density lipoprotein; HDL, high density lipoprotein; BP, blood pressure; ^a^ These assessments were performed in cases in which total cholesterol was ≥ 200 mg/dL and after fasting for 6 h or total cholesterol ≥ 250 mg/dL regardless of fasting hours. ^b^Affected territories include bilateral carotid (common, bifurcation, internal, and external) and femoral (common and superficial) arteries.

**Table 2:** Baseline clinical and metabolic characteristics, and atheromatous status of participants based on the new definition of obesity (BMI ≥30 kg/m² plus BMI 25–30 kg/m² with a WtHR ≥ 0.5 and at least one obesity-related health condition) proposed by EASO.

Characteristic	No obesity group	New definition of obesity	*P*
*n*	2.355 (28.3)	5.975 (71.7)	–
Women (%)	54.0	49.3	< 0.001
Age (yrs)	55.5 (51.5–60.5)	58.5 (53.5–63.5)	< 0.001
BMI (kg/m²)	23.9 (22.3–25.0)	30.9 (27.7–33.1)	< 0.001
Waist to Height Ratio	0.55 (0.51–0.58)	0.65 (0.59–0.68)	< 0.001
Prediabetes (%)	15.8	41.9	< 0.001
Glycaemia (mg/dl)	92.0 (86.0–99.0)	97.0 (90.0–106.0)	< 0.001
Dyslipidaemia (%)	36.9	57.3	< 0.001
Total cholesterol (mg/dl)	215.0 (191.0–239.0)	215.5 (190.0–240.0)	0.507
LDL-cholesterol ^a^ (mg/dl)	132.4 (111.2–153.2)	131.6 (112.0–154.0)	0.695
HDL-cholesterol ^a^ (mg/dl)	60.0 (51.0–70.0)	54.0 (47.0–62.0)	< 0.001
Triglycerides ^a^ (mg/dl)	96.0 (74.0–132.0)	126.0 (94.0–173.0)	< 0.001
Lipid-lowering agents (%)	10.1	21.2	< 0.001
Hypertension (%)	16.3	49.3	< 0.001
Systolic BP (mm Hg)	124.0 (114.0–135.0)	133.0 (122.0–144.0)	< 0.001
Diastolic BP (mm Hg)	78.0 (72.0–84.0)	83.0 (77.0–89.0)	< 0.001
Pulse Pressure (mm Hg)	45.0 (39.0–53.0)	49.0 (42.0–58.0)	< 0.001
Antihypertensives (%)	16.9	49.9	< 0.001
Current or former smoker (%)	63.7	33.9	< 0.001
Characteristics of atheromatous disease			
Presence of any plaque ^b^ (%)	68.7	72.9	< 0.001
Number of affected territories, n	1.9 (0.0–3.0)	2.1 (0.0–3.0)	< 0.001
Total plaque area (cm²)	0.8 (0.2–1.1)	0.7 (0.2–1.0)	0.834
Carotid territory affected ^b^ (%)	46.	52.1	< 0.001
Number of carotid plaques, n	0.9 (0.0–2.0)	1.1 (0.0–2.0)	< 0.001
Carotid plaque area (cm²)	0.3 (0.1–0.4)	0.3 (0.1–0.4)	0.938
Femoral territory affected ^b^ (%)	53.7	55.8	0.083
Number of femoral plaques	1.0 (0.0–2.0)	1.0 (0.0–2.0)	0.273
Femoral plaque area (cm²)	0.7 (0.2–1.0)	0.7 (0.3–1.0)	0.366

Data are expressed as median (interquartile range), *n* or percentage. BMI, body mass index; EASO, European Association for the Study of Obesity; LDL, low density lipoprotein; HDL, high density lipoprotein; BP, blood pressure; ^a^ These assessments were performed in cases in which total cholesterol was ≥ 200 mg/dl and after fasting for 6 h or total cholesterol ≥ 250 mg/dl regardless of fasting hours. ^b^Affected territories include bilateral carotid (common, bifurcation, internal, and external) and femoral (common and superficial) arteries.

**Table 3:** Baseline clinical and metabolic characteristics, and atheromatous status of participants adding to the classical definition of obesity (BMI ≥ 30 kg/m²) those participants with overweight (BMI 25–30 kg/m²) and abdominal obesity (WtHR ≥ 0.5).

Characteristic	No obesity group	New definition of Obesity without including comorbidities in BMI 25–30 kg/m^2^	*p*
*n* (%)	1,762 (21.1)	6,568 (78.8)	–
Women (%)	60.5	48.0	< 0.001
Age (years)	56.5 (52.5–61.5)	58.5 (53.5–63.5)	< 0.001
BMI (kg/m²)	23.3 (21.8–24.3)	29.7 (27.4–32.8)	< 0.001
Waist to height ratio	0.5 (0.5–0.6)	0.6 (0.6–0.7)	< 0.001
Prediabetes (%)	20.9	38.1	< 0.001
Glycaemia (mg/dl)	91.0 (85.0–98.0)	97.0 (90.0–105.0)	< 0.001
Dyslipidaemia (%)	49.3	52.2	0.034
Total cholesterol (mg/dl)	219.0 (193.0–244.0)	214.0 (190.0–239.0)	0.029
LDL-cholesterol ^a^ (mg/dl)	133.3 (112.1–155.6)	131.4 (111.6–153.6)	0.296
HDL-cholesterol ^a^ (mg/dl)	62.0 (53.0–72.0)	54.0 (47.0–62.0)	< 0.001
Triglycerides ^a^ (mg/dl)	95.0 (73.0–128.0)	124.0 (91.0–172.0)	< 0.001
Lipid-lowering agents (%)	13.4	19.4	< 0.001
Hypertension (%)	21.8	44.9	< 0.001
Systolic BP (mm Hg)	124 (113–135)	132 (121–143)	< 0.001
Diastolic BP (mm Hg)	77 (71–84)	83 (77–89)	< 0.001
Pulse pressure (mm Hg)	46 (39–54)	48 (41.5–57)	< 0.001
Antihypertensives (%)	21.9	45.6	< 0.001
Current or former smoker (%)	57.2	38.3	< 0.001
Characteristics of atheromatous disease			
Presence of any plaque ^b^ (%)	68.8	72.5	0.002
Number of affected territories, *n*	1.9 (0.0–3.0)	2.1 (0.0–3.0)	0.006
Total plaque area, (cm²)	0.5 (0.2–1.1)	0.5 (0.2–1.1)	0.426
Carotid territory affected ^b^ (%)	47.4	51.3	0.004
Number of carotid plaques, *n*	1.0 (0.0–2.0)	1.1 (0.0–2.0)	0.009
Carotid plaque area, (cm²)	0.2 (0.1–0.4)	0.2 (0.1–0.4)	0.806
Femoral territory affected ^b^ (%)	53.3	55.7	0.066
Number of femoral plaques, *n*	1.0 (0.0–2.0)	1.0 (0.0–2.0)	0.158
Femoral plaque area, (cm²)	0.5 (0.3–1.0)	0.5 (0.2–1.0)	0.366

Data are expressed as median (interquartile range), n or percentage. BMI, body mass index; LDL, low density lipoprotein; HDL, high density lipoprotein; BP, blood pressure; ^a^ These assessments were performed in cases in which total cholesterol was ≥ 200 mg/dL and after fasting for 6 h or total cholesterol ≥ 250 mg/dL regardless of fasting hours. ^b^Affected territories include bilateral carotid (common, bifurcation, internal, and external) and femoral (common and superficial) arteries.

Regarding atherosclerotic disease, the classical definition of obesity showed no statistically significant differences in the prevalence of atheromatous disease across territories compared with participants without obesity. In contrast, the new definition identified a higher prevalence of plaques in the obesity group (72.9% vs. 68.6%; *p* < 0.001), with more affected territories (2.1 vs. 1.9; *p* < 0.001), with the carotid being more frequent (52.1% vs. 46.3%; *p* < 0.001) (**Tables 1**, **2**). When only subjects with abdominal obesity (WtHR ≥ 0.5) but no comorbidities were included, similar patterns of increased atherosclerotic burden were observed (**Table 3**).

When multivariable logistic regression was performed to examine the factors independently associated with SAD, the new EASO obesity criteria emerged as an independent predictor of overall plaque presence (OR 1.54, 95% CI 1.22–1.94, *p* < 0.001) and of femoral plaque (OR 1.36, 95% CI 1.10–1.68, *p* = 0.004), but not of carotid plaque (*p* = 0.290) (**Table 4**). Similar results were observed when this definition was replaced by only abdominal obesity (WtHR ≥ 0.5) (**Table 5**). In contrast, a BMI ≥ 30 kg/m^2^ alone was not an independent predictor of SAD, neither overall nor in any vascular territory (**Table 6**).

**Table 4:** Stepwise logistic regression model to assess factors independently associated with the presence of any plaque, plaque in carotid territories, or plaque in femoral territories according to the new definition of obesity proposed by EASO.

Factor	Presence (yes/no) of any plaque		Presence (yes/no) of any carotid plaque		Presence (yes/no) of any femoral plaque	
	Odds ratio (95% CI)	*p*	Odds ratio (95% CI)	*p*	Odds ratio (95% CI)	*P*
Sex (women)	0.40 (0.31–0.53)	< 0.001	0.47 (0.41–0.54)	< 0.001	0.43 (0.34–0.55)	< 0.001
Age (years)	1.10 (1.08–1.11)	< 0.001	1.08 (1.06–1.09)	< 0.001	1.07 (1.06–1.09)	< 0.001
Hypertension (yes/no)	1.39 (1.18–1.64)	< 0.001	1.31 (1.14–1.50)	< 0.001	1.48 (1.28–1.72)	< 0.001
Pulse pressure (mm Hg)	1.02 (1.01–1.03)	< 0.001	1.02 (1.02–1.03)	< 0.001	1.00 (1.00–1.01)	0.017
Prediabetes (yes/no)	–	0.366	1.22 (1.07–1.40)	0.004	–	0.666
Total cholesterol (mg/dl)	1.00 (1.00–1.00)	< 0.001	1.00 (1.00–1.00)	< 0.001	1.00 (1.00–1.00)	< 0.001
Triglycerides (mg/dl)	–	0.799	–	0.706	1.00 (1.00–1.00)	0.046
New obesity definition (yes/no)	1.54 (1.22–1.94)	< 0.001	–	0.291	1.36 (1.10–1.68)	0.004
Smoking (yes/no)	3.19 (2.67–3.81)	< 0.001	1.76 (1.52–2.03)	< 0.001	3.58 (3.06–4.19)	< 0.001
Test of fit Hosmer–Lemeshow	–	0.702	–	0.372	–	0.796
Area under de ROC curve	–	0.722	–	0.676	–	0.716

EASO, European Association for the Study of Obesity; ROC, Receiver Operating Characteristic.

**Table 5:** Stepwise logistic regression model to detect factors independently associated with the presence of any plaque, plaque in carotid territories, or plaque in femoral territories according to the classical definition of obesity.

Factor	Presence (yes/no) of any plaque		Presence (yes/no) of any carotid plaque		Presence (yes/no) of any femoral plaque	
	Odds ratio (95% CI)	*kp*	Odds ratio (95% CI)	*p*	Odds ratio (95% CI)	*P*
Sex (women)	0.31 (0.26–0.36)	< 0.001	0.47 (0.41–0.54)	< 0.001	0.33 (0.28–0.38)	< 0.001
Age (years)	1.10 (1.01–1.11)	< 0.001	1.08 (1.06–1.09)	< 0.001	1.07 (1.06–1.09)	< 0.001
Hypertension (yes/no)	1.44 (1.23–1.70)	< 0.001	1.31 (1.14–1.50)	< 0.001	1.50 (1.30–1.73)	< 0.001
Pulse pressure (mm Hg)	1.02 (1.01–1.02)	< 0.001	1.02 (1.02–1.03)	< 0.001	1.00 (1.00–1.01)	0.020
Prediabetes (yes/no)	–	0.179	1.22 (1.07–1.40)	0.004	–	0.438
Total cholesterol (mg/dl)	1.00 (1.00–1.00)	< 0.001	1.00 (1.00–1.00)	< 0.001	1.00 (1.00–1.00)	< 0.001
Triglycerides (mg/dl)	–	0.685	–	0.721	1.00 (0.99–1.01)	0.057
Classical obesity definition (yes/no)	–	0.948	–	0.316	–	0.130
Smoking (yes/no)	3.00 (2.53–3.60)	< 0.001	1.76 (1.52–2.03)	< 0.001	3.49 (3.00–4.07)	< 0.001
Test of fit Hosmer–Lemeshow	–	0.180	–	0.372	–	0.422
Area under de ROC curve	–	0.719	–	0.676	–	0.714

ROC, receiver operating characteristic.

**Table 6:** Stepwise logistic regression model to assess factors independently associated with the presence of any plaque, plaque in carotid territories, or plaque in femoral territories when individuals with overweight and abdominal obesity (WtHR ≥ 0.5) were included in the model.

Factor	Presence (yes/no) of any plaque		Presence (yes/no) of any carotid plaque		Presence (yes/no) of any femoral plaque	
	Odds ratio (95% CI)	*p*	Odds ratio (95% CI)	*p*	Odds ratio (95% CI)	*p*
Sex (women)	0.38 (0.29–0.50)	< 0.001	0.47 (0.41–0.54)	< 0.001	0.43 (0.33–0.55)	< 0.001
Age (years)	1.10 (1.08–1.11)	< 0.001	1.08 (1.06–1.09)	< 0.001	1.07 (1.06–1.09)	< 0.001
Hypertension (yes/no)	1.44 (1.22–1.69)	< 0.001	1.31 (1.14–1.50)	< 0.001	1.52 (1.31–1.76)	< 0.001
Pulse pressure (mm Hg)	1.02 (1.01–1.03)	< 0.001	1.02 (1.02–1.03)	< 0.001	1.00 (1.00–1.01)	0.017
Prediabetes (yes/no)	–	0.193	1.22 (1.07–1.40)	0.004	–	0.459
Total cholesterol (mg/dl)	1.00 (1.00–1.00)	< 0.001	1.00 (1.00–1.00)	< 0.001	1.00 (1.00–1.00)	< 0.001
Triglycerides (mg/dl)	–	0.707	–	0.736	1.00 (1.00–1.00)	0.043
Abdominal obesity (yes/no)	1.41 (1.10–1.81)	0.006	–	0.344	1.32 (1.05–1.66)	0.016
Smoking (yes/no)	3.06 (2.57–3.64)	< 0.001	1.76 (1.52–2.03)	< 0.001	3.49 (2.99–4.07)	< 0.001
Test of fit Hosmer–Lemeshow	–	0.352	–	0.371	–	0.314
Area under de ROC curve	–	0.721	–	0.676	–	0.713

## Discussion

To our knowledge, this is the first study to evaluate the new criteria for the diagnosis of obesity and its association with the presence of SAD in a large cohort of participants with low to moderate cardiovascular risk. Our findings demonstrate that these updated criteria offer greater predictive capacity for atherosclerotic burden by emphasizing central adiposity over BMI alone. These results align with previous studies that have highlighted the significance of central adiposity, particularly VAT, in the development of cardiovascular and atheromatous diseases, rather than overall body mass (2, 8, 9). Therefore, our analysis suggests that adiposity may play a different role in the development of SAD across different vascular territories, with a higher impact on femoral atherosclerosis.

VAT is a known metabolically active tissue that contributes to cardiovascular risk through multiple mechanisms. First, VAT is highly proinflammatory, producing adipokines such as leptin, TNF-α, and IL-6, which promote endothelial dysfunction, inflammation, and insulin resistance. These factors collectively lead to atherosclerosis and CVD (10, 11). Secondly, the “portal hypothesis” suggests that visceral fat releases free fatty acids directly into the portal circulation, leading to an enhancement of hepatic insulin resistance, hepatic glucose production, and dyslipidemia, also key risk factors for CVD (12). In this context, the American Heart Association (AHA) also emphasizes that visceral adiposity is strongly linked to metabolic complications such as hypertriglyceridemia, hyperinsulinemia, and glucose intolerance, making it a more accurate predictor of cardiovascular risk than overall body mass (12). Moreover, prospective studies have demonstrated that visceral fat is independently associated with incident CVD and all-cause mortality, whereas subcutaneous fat does not exhibit the same correlation (13). For instance, the Multi-Ethnic Study of Atherosclerosis found that higher levels of visceral fat were significantly associated with an increased risk of coronary heart disease (13).

In this context, the WtHR has been used as a criterion for better characterizing central adiposity and has proven to be a strong predictor of CVD. Supporting this, our data shows that individuals with overweight and abdominal adiposity, regardless of associated comorbidities, present a higher risk of presenting SAD, with a stronger association observed in the femoral territories. Similarly, a prospective cohort study from the UK Biobank demonstrated that WtHR was associated with ischemic CVD, heart attacks, and ischemic strokes, independently of overall body weight (24). Moreover, the AHA considers WtHR a superior predictor of CVD compared to BMI (12). In line with these findings, a meta-analysis of 31 prospective studies further reinforces the association between higher WtHR and a significantly increased risk of CVD (25). Reinforcing this evidence and moving toward a paradigm shift in obesity management, the EASO has recently introduced a new framework that aligns obesity diagnosis with chronic disease standards (2). This change is based on the recognition that the diagnostic criteria for obesity should extend beyond BMI, acknowledging that body fat distribution significantly impacts health (2). More specifically, this new framework includes individuals with a lower BMI (≥25–30 kg/m^2^) but increased abdominal fat accumulation and the presence of any medical, functional, or psychological impairments or complications in the definition of obesity, hence reducing the risk of undertreatment in this particular group of patients compared to the current BMI-based definition (2). Our findings support this new position statement, as only patients meeting the new obesity criteria exhibited a worse glycemic and hypertensive profile, required more prescriptions for antihypertensive and lipid-lowering therapies, and showed an increased prevalence of SAD.

Our study provides the first evidence regarding the significant association between the new obesity definition and the presence of atheroma plaques in femoral artery territory. This finding is particularly relevant as atherosclerosis is a multifaceted disease influenced by factors such as hemodynamic forces, genetic predisposition, sex, immune status, oxidative stress, and chronic low-grade inflammation (26). While the specific pathogenic mechanisms underlying plaque formation and their variable impact across vascular territories are not yet fully understood, evidence suggests that the localization of atherosclerotic plaques plays a pivotal role in disease progression (14, 15).

Taking into consideration studies on femoral plaque burden, such as that by Vaudo et al., evidence suggests that patients with metabolic syndrome exhibit increased intima-media thickness (IMT) at the femoral site compared to healthy controls. Their findings indicate that femoral IMT is directly associated with LDL cholesterol, triglycerides, and glycemia (27). Similarly, the Progression of Early Subclinical Atherosclerosis (PESA) study demonstrated a significant association between femoral plaque burden and factors such as age, sex, smoking, and dyslipidemia, with comparable results observed in the Aragón Worker’s Heath Study (AWHS) (28, 29). Conversely, our results also suggest that the impact of obesity on the development of atheromatous disease in carotid territories may be more associated with other CV risk factors, such as hypertension, prediabetes, or smoking, which would potentially play a more significant role in its development. The proximity of femoral arteries to abdominal visceral adipose tissue (VAT) may further contribute to this process through paracrine signaling mechanisms that favor the formation of dense, calcified plaques (30–33). Imaging studies using magnetic resonance have also shown that femoral plaques typically contain smaller necrotic cores and fewer intraplaque hemorrhages compared to carotid plaques, further highlighting their distinct pathophysiological profile (34).

The high prevalence of obesity found in our study merits further comments. Our data show that 37.2% of individuals with low-to-moderate CV risk recruited in the ILERVAS cohort had a BMI ≥30 kg/m², nearly double the prevalence reported in Spain (overall prevalences of obesity and overweight of 18.7% and 55.8%, respectively) (35). These elevated rates can likely be attributed to the inclusion criteria, which required participants to be over 45 years of age and present at least one CV risk factor—obesity, as defined by BMI, being one of them. The prevalence rose above 70% when we included individuals with overweight and abdominal adiposity, regardless of the presence of obesity-related comorbidities. Apart from the characteristics of our study, the further increase of obesity following the new EASO criteria, together with the elevated prevalence of SAD revealed in this population, opens up a new scenario not only in epidemiological terms but also in the sustainability of healthcare systems due to the huge economic burden of the associated treatments.

Our study has several limitations that warrant discussion. First, its cross-sectional design prevents us from establishing causal relationships. Second, the assessment of atherosclerosis was confined to the carotid and femoral territories, excluding other vascular regions such as the aorta or coronary arteries, which are also relevant to measuring cardiovascular risk. Additionally, our study exclusively included individuals with at least one cardiovascular risk factor, which may partly explain the high prevalence of SAD observed. However, when including participants with overweight and abdominal obesity without associated obesity-related comorbidities, the results remained consistent, further highlighting the role of VAT in the development of CVD. Finally, a complete lipid profile was only performed in patients with total cholesterol ≥ 200 mg/dl, which may have led to missing atherogenic dyslipidemia in individuals with normal LDL or total cholesterol. Despite these limitations, an important strength of our study is the inclusion of a large, well-characterized cohort of individuals at low-to-moderate cardiovascular risk without established cardiovascular disease.

In summary, this study underscores the critical role of VAT in influencing atheromatosis, particularly in femoral territories, which may have significant implications for CV risk assessment and clinical management. Our results also suggest that the shifting from BMI toward the new obesity definition that includes WtHR, which represents a move from a weight-centered approach to one based on visceral adiposity, leads to earlier identification of SAD, and, therefore, more targeted interventions for individuals at risk of developing serious cardiovascular conditions can be implemented.

## ILERVAS Project collaborators

Carolina López-Cano, Eva Miquel, Marta Ortega, Ferran Barbé, Jordi de Batlle, Silvia Barril, Manuel Portero-Otín, Mariona Jové, Josep Franch-Nadal, Esmeralda Castelblanco, Pere Godoy, Montse Martinez- Alonso, and Cristina Farràs.

## Data Availability

The raw data supporting the conclusions of this article will be made available by the authors, without undue reservation.
